# Simple one-step synthesis of urchin-like Fe–Mn nanostructures via statistical design and their effects on the morphology

**DOI:** 10.1038/s41598-022-23381-9

**Published:** 2022-11-12

**Authors:** Saeid Naeinimohammadi, Ahmad Rahbar-Kelishami

**Affiliations:** Faculty of Chemical, Petroleum and Gas Engineering, Iran University of Science and Technology (IUST), Narmak, Tehran, Iran

**Keywords:** Chemical engineering, Environmental chemistry

## Abstract

In the current investigation, a new urchin-like nanostructure using an authorized one-pot precipitation technique was synthesized using Taguchi statistical design. The effect of factors, including the concentration of FeSO_4_⋅7H_2_O, KMnO_4_, NaOH, and reaction temperature, on the diameter-to-length ratio of the nanoneedles and the regularity or irregularity morphology of other samples, was investigated. KMnO_4_ and FeSO_4_⋅7H_2_O, with a contribution of 32.62% and 30.9%, had the most substantial effect on the nanoneedles' diameter. The surface morphology and chemical composition of the as-prepared samples were studied using field emission scanning electron microscopy, X-ray diffraction, and energy dispersive spectroscopy (EDX) analyses. The length and needle diameter was obtained 140 and 17 nm, respectively. The result of BET analysis for the highest and the lowest value of D/L shows that the smallest ratio of diameter to length has a high specific surface area. The results show that sample S4 has a particular surface area of 74 m^2^/g, much more than S3 (25 m^2^/g). The estimated crystallite size in synthesized S3 and S4 samples are 41.64 and 26.49 nm, respectively.

## Introduction

The urchin-like nanostructures of double oxides have been drawn into significant consideration. They can definitively make strides in reactivity owing to their extended surface ranges and composite material such as Fe–Mn. These materials have received considerable immersion because of chemical and physical estates. On the contrary, Fe–Mn, as a powder with a nanoscale dimension, presents great potential for its application in different processes. Generally, prickly structures, especially urchin-like nanostructures, are used increasingly in water treatment, medicine, drug delivery, the food industry, oil/water separation, electrochemical sensors, and catalysts^1–7^.

Research within the blend technique of nanomaterials is primarily situated toward controlling their shape, measure, and composition. Each of these components may be a key calculate in deciding a key calculate in determining the properties of materials that lead to distinctive, innovative applications^8,9^. Nanosystems have come to colossal consideration over time. The elemental component of nanosystems is nanoparticles. The size of nanomaterials is between 1 and 100 nm and is included metal oxides, carbon, metal, metal, or natural material. The materials at the nonmetric size, compared to the other materials at higher scales, demonstrate unique biological, physical, and chemical characteristics^10–12^. This knowledge is caused by a comparatively bigger surface area to the volume, expanded reactivity or toughness in a chemical handle, heightened mechanical strength, etc.

The classification of nanostructure materials (NSMs) that have been established was essential. As a subject of nanotechnology, nanomaterials have low structural materials surrounding nanoscale size structural units at a minimum in one management. Gleiter provided the first nanomaterial category in 1995, and Skorokhod was promoted in 2001^13^. Nevertheless, the provided plan by Gleiter and Skorokhod was examined entirely because 0-dimensional (0D), 1-dimensional (1D), 2-dimensional (2D), and 3-dimensional (3D) constructions such as nanotubes, fullerenes, and nan flowers were not captured within report^14^. Hence, the category plan was altered by including 0D,1D, 2D, and 3D nanostructures, as Pokropivny and Skorokhod reported for nanomaterials^15^. Unique properties such as great specific surface area and 3D nonmaterial have enticed substantial studies in the last decade^16^. The sizes, shapes, proportions, and nanomaterial embryologists affect the manner of nanostructure materials. Consequently, to arrange 3D nanostructures, materials with a restrained morphology and structure were attracted. Furthermore, the applications of 3D nanostructures are significant in charismatic material, catalysis, and electrode material for batteries^13,15,17–30^. Additionally, the 3D NSMs have as of late pulled in seriously investigate interface since the nanomaterials have a greater surface range and supply sufficient adsorption destinations for all included atoms in a little space^31^. For example, Zheng synthesized urchin-like trimanganes tetraoxide particles with oxidase-like activity for glutathione detection through a simple and gentle method^32^. He et al. prepared sea-urchin-like carbon/ZnO microstructures using a novel low-temperature hydrothermal method for enhancing photocatalytic activity. The average diameter of nanoneedles was 40–50 nm^33^. Zhou et al. created the 3D urchin-like TiO_2_-reduced graphene micro/nanostructure composite through two forms. The adjoining cross section was 0.32 nm. The BET parameters were obtained from the N_2_ adsorption–desorption isotherm. The particular surface zone comes to up to 117.56 m^2^/g^34^. Liu et al. synthesized the urchin-like structure of bimetallic phosphides by a simple solvothermal method. The results of scanning electron microscopy showed the length and diameter of nanorods formed on the surface were about 300–500 nm and 20 nm, respectively^35^. Chen et al. reported synthesizing urchin-like NiCo_2_O_4_ hollow nanospheres based on a special stepwise co-precipitation method in 2018^36^. Song et al. synthesized urchin-like AlOOH microspheres with large specific surface area by a one-pot chemical-induced solvothermal method^37^. Abraham et al. 2018 synthesized nanourchin-structured α-MnO_2_ for the application of ultrasonic-assisted adsorptive removal of cationic dyes synthesis. The diameter of nanoneedles was 20 nm^38^.

Nanourchin-like FeMn_x_O was synthesized through the simple chemical method by Zhong in 2016. The field emission scanning electron result showed that the nanostructure contains an amorphous Fe–Mn double oxides essence and a regular FeOOH nanoneedle shell. The specific surface area of synthesized material was reported to be about 142 m^2^/g^39^. In this study, the effect of fundamental factors, including the concentration of FeSO_4_.7H_2_O, KMnO_4_, NaOH, and reaction temperature (70–100 °C) on the diameter to length of nanoneedles, regularity or irregularity morphology of all samples were investigated.

## Experimental

### Materials

The details of the materials are listed in Table 1. All chemicals were of analytical grade and purchased from Merck Company without further purification.

**Table 1:** Materials used in the present study.

Reagent	EC. index no	Formula	Molecular weight (g/mol )	Manufacturer
Ferroussulfate heptahydrate	026-003-01-4	FeSO_4_⋅7H_2_O	278.02	Merck Co., German
Potassium permanganate	025-002-00-9	KMnO_4_	158.03	Merck Co., German
Sodium hydroxide	011-002-00-6	NaOH	40	Merck Co., German

### Taguchi statistical design

Design of the experiment (DOE) as a quantifiable approach not because it diminishes the number of the tests to induce the perfect conditions but also chooses the impact of the factors^40^. In addition, DOE responds with fewer tests as a competent instrument to accomplish perfect conditions. The Taguchi statistical design is one of the foremost common test plan strategies. DOE can incorporate taking after steps: (a) Select the objective work or reaction variable, (b) Classification of variable, (c) Deciding levels of the compelling components, and (d) Test of affectability with the assistance of an orthogonal cluster. To explore the concentration of FeSO_4_⋅7H_2_O (factor A), the concentration of KMnO_4_ (factor B), the concentration of NaOH (factor C), and reaction temperature (factor D), Taguchi L_9_ orthogonal were investigated for the ratio of diameter to length of nanoneedles as a response, which showing in Tables 2 and 3^37^.

**Table 2:** Factors and levels.

Value	Level	Symbol	Parameters (unit)
180	A_1_	A	Concentration of FeSO_4_⋅7H_2_O (mM)
225	A_2_		
270	A_3_		
59	B_1_	B	Concentration of KMnO_4_ (mM)
75	B_2_		
90	B_3_		
4	C_1_	C	Concentration of NaOH (M)
5	C_2_		
6	C_3_		
70	D_1_	D	Temperature (°C)
85	D_2_		
100	D_3_

**Table 3:** Taguchi L_9_ ortoghonal array.

Sample	Factors and levels			
	A	B	C	D
1	180	59	4	70
2	180	75	5	85
3	180	90	6	100
4	225	59	5	100
5	225	75	6	70
6	225	90	4	85
7	270	59	6	85
8	270	75	4	100
9	270	90	5	70

The precipitation method was utilized to synthesize Fe–Mn parallel oxide powder. According to the Taguchi design, FeSO_4_⋅7H_2_O and KMnO_4_ were independently solved in 100 mL of deionized water. The arranged KMnO_4_ solving was heated to definite temperature. After that, FeSO4 arrangement continuously included into the KMnO4 beneath energetic attractive blending was moderately added into the KMnO4 solving under forceful, charismatic animated 13 mL of NaOH was at that point included dripping to the blend. After 5 min, the response was turned off and the blended arrangement was cooled to the surrounding temperature. The accelerates were possessed and washed occasionally with deionized water to expel the pollution. After washing, the item was dried and stored at 80 °C to steady weight. The evaporated item was pulverized and put away in the drier for advances to apply.

### Characterization

Fourier-transform infrared spectroscopy (FTIR) analysis was used to investigate functional groups using Perkinelmer-USA. X-ray diffraction plans of samples were recorded interior 2θ of 10°–80° by using Bruker XRD Model Advance. The surface area of these textures was intercepted by Belsorp (Mini Finted-Japan) using N_2_ as adsorbate at 77 K. Also, Barret-Joyner-Halenda (BJH) method was employed to determine pore size dispersal and average pore width. Field-emission scanning electron amplifying (FESEM KYKY-EM8000F-HV-15 kV-China and ZEISS SIGMA 300-German) qualified with EDAX system was utilized for magnifying electron microscopy examination to study the surface analysis textural structures of samples.

## Results and discussion

### Taguchi analysis

Table 5 shows the ANOVA analysis of the Taguchi L_9_ orthogonal array responses. One of the most important information outputs from ANOVA analysis is the significance or non-significance of factors in statistical analysis^41–43^. The impact of each calculation was distinctive from the other. Subsequently, D and C are more vital than the other two variables. Reliable with other researchers' thoughts^38,39^. The commitment percent of each calculated from the most elevated to the least esteem on the proportion of D/L as the inspection reaction profitable for B, A, C, and D were 32.13, 30.90, 20.02, and 16.95%, respectively.

**Figure 1:**
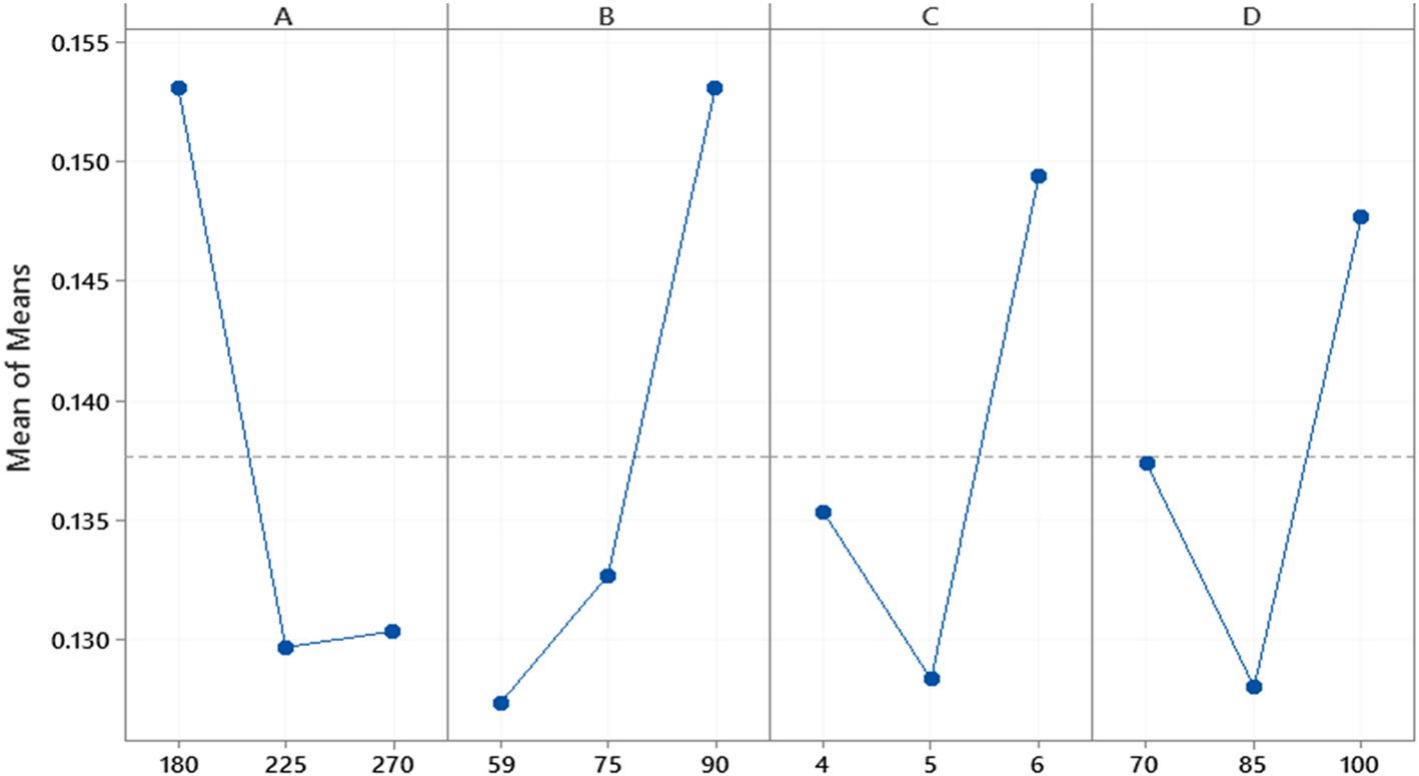
Main effects plot for means.

Moreover, to assess each factor's ideal levels, the factors’ primary main effect plots were connected (Fig. 1). In this strategy, the more minor reactions from each figure level are calculated to get each factor’s fundamental impact of nanoneedles reconditions^39^. The Taguchi method assessed the impact of different components within the amalgamation of nanomaterial on the proportion of distance across to length (Table 4). Comparing the comes about appears that the calculate impacts recorded from the most noteworthy are B, A, C, and D, respectively. It can be seen from the most impacts plot that the anticipated ideal conditions for the least D/L will be gotten at 225 mM of FeSO_4_⋅7H_2_O solution, 59 mM of KMnO_4_, and 5 M NaOH^32,44^.

**Table 4:** The ratio of diameter to length measurements of Taguchi L_9_.

Sample	Factors and levels				Response (D/L)
S1	180	59	4	70	0.14
S2	180	75	5	85	0.125
S3	180	90	6	100	0.19
S4	225	59	6	100	0.12
S5	225	75	5	75	0.13
S6	225	90	4	85	0.133
S7	270	59	6	85	0.122
S8	270	75	4	100	0.133
S9	270	90	5	70	0.136

According to Table 5, DF (degree of freedom) shows the examination employments that date to estimate the worth of unknown culture parameters. The number of observations in the sample determines the total DF. Sequential sums of squares (Seq SS) are measures of variation for different model components. Minitab software uses the Seq SS to calculate the P-value and the R^2^ statistic. Adjusted sums of squares (Adj SS) are measures of variation for diverse elements of the model. Minitab separates the sums of squares into different components that portray the variety due to a distinctive source. Adjusted mean squares (Adj MS) measure how much a term or a model explains.

**Table 5:** Analysis of variance for means.

Source	DF (degree of freedom)	Seq SS (sequential sums of squares)	Adj SS (adjusted sum of squares)	Adj MS (adjusted mean squares)
A	2	0.000955	0.000955	0.000477
B	2	0.0013930	0.001393	0.000696
C	2	0.000620	0.000620	0.000310
D	2	0.000575	0.000575	0.000287
Residual	0	–	–	–
Error	–	–	–	–
Total	8	0.003543		

### Nanourchin-like Fe–Mn parallel oxide nanoneedles growth mechanism

**Figure 2:**
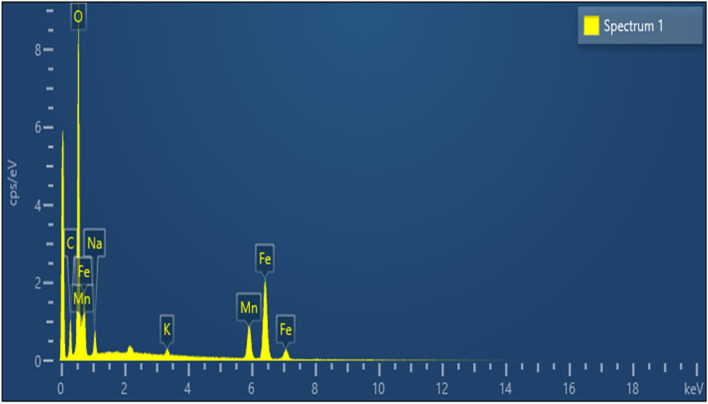
EDX surface analysis of sample S4.

Nanourchin-like Fe–Mn was synthesized through a one-step reaction without presenting any surfactant. The transition of Fe–Mn double oxides was explored through FESEM analysis. Energy dispersive X-ray and X-ray diffraction investigation of the accelerates gathered at the diverse condition of amalgamation. The comparing EDX investigation of sample S4 (Fig. 2 and Table 6) appeared that these nanoparticles are created of Fe, Mn, and O components, and the molar proportion is smaller than the Fe/Mn molar proportion within the introductory measurements, demonstrating that a few of Fe particle may still be displayed within the supernatant.

**Table 6:** EDX analysis of sample S4.

Element	Line Type	Weight %	Weight % sigma	Atomic %
C	K series	14.93	0.54	26.48
O	K series	41.65	0.39	55.44
Mn	K series	10.65	0.22	4.13
Fe	K series	29.69	0.35	11.32
Na	K series	2.49	0.10	2.30
K	K series	0.58	0.05	0.32

**Figure 3:**
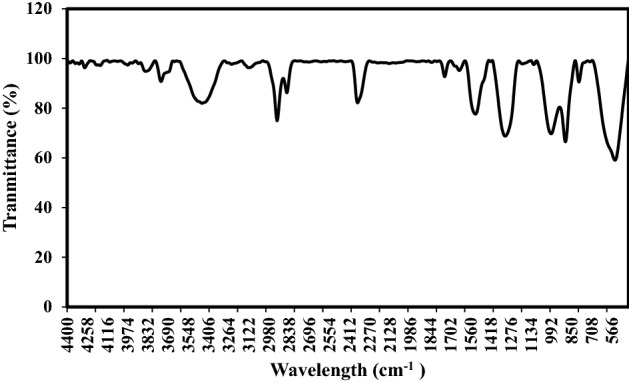
FTIR spectra of S3.

**Figure 4:**
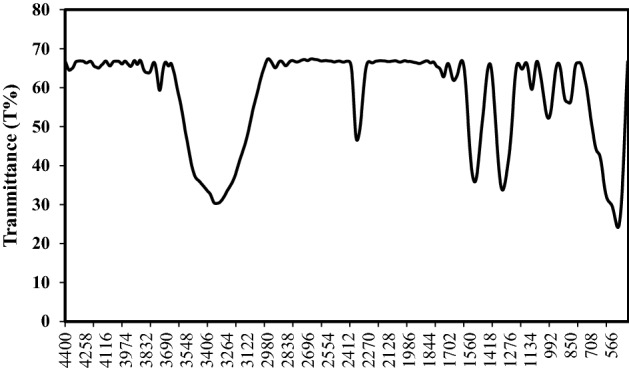
FTIR spectra of S4.

The surface chemistries of the Fe–Mn nanourchin-like (S3 and S4) were analyzed utilizing the FTIR processor, and the resulting spectrogram is manifested in Figs. 3 and 4. A broad and sharp peak can be perceived within the extend from 3200 to 3450 cm^−1^ and was gathered to start from –OH stretching vibration of hydroxyl groups^45,46^. The foremost vital signal at 580, 960, and 1550 cm^−1^ corresponds to MnO_2_, FeOH, and Fe_2_O_3_, respectively.

**Figure 5:**
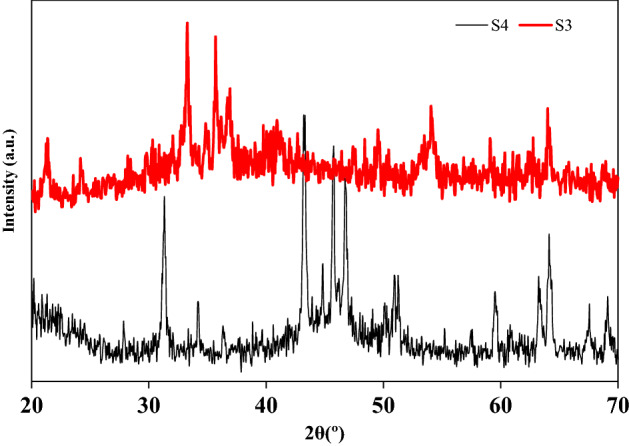
XRD patterns of samples S3 and S4.

The composition and crystalline structure of synthesized Fe–Mn double oxides (sample S3, S4) were identified by XRD technique with Cu-Ka radiation (λ = 0.154 nm) (Fig. 5). The diffraction patterns were taken in 2θ mode in the 10°–80° range. The XRD patterns of samples S3 and S_4_ display a few clear diffraction crests coordinating the stage of FeOOH (01-81-0464). Thus, the nanoneedles in the Fe–Mn composite were FeOOH^35,47^. Further, more obvious diffraction crystalline peaks of manganese oxide and Fe_2_O_3_ are detected. The crystallite degree was assessed by Scherer֜ s equation (Eq. 1).

1 $${\text{D}}_{{{\text{hkl}}}} \, = \,{\text{c}}\lambda /\beta {\text{Cos}}\theta$$

where c is constant (−0.89), λ is the wavelength of the radiation utilized (λ_Cu_ = 0.154 nm), θ is the diffraction point in degrees, and β is the overall width at half most prominent of the spreading point most extraordinary in radians (FWHM). The crystallite assessment inside the Fe–Mn double oxides particles for S3 and S4 samples were obtained 41.64 and 26.49 nm, respectively^44^.

**Figure 6:**
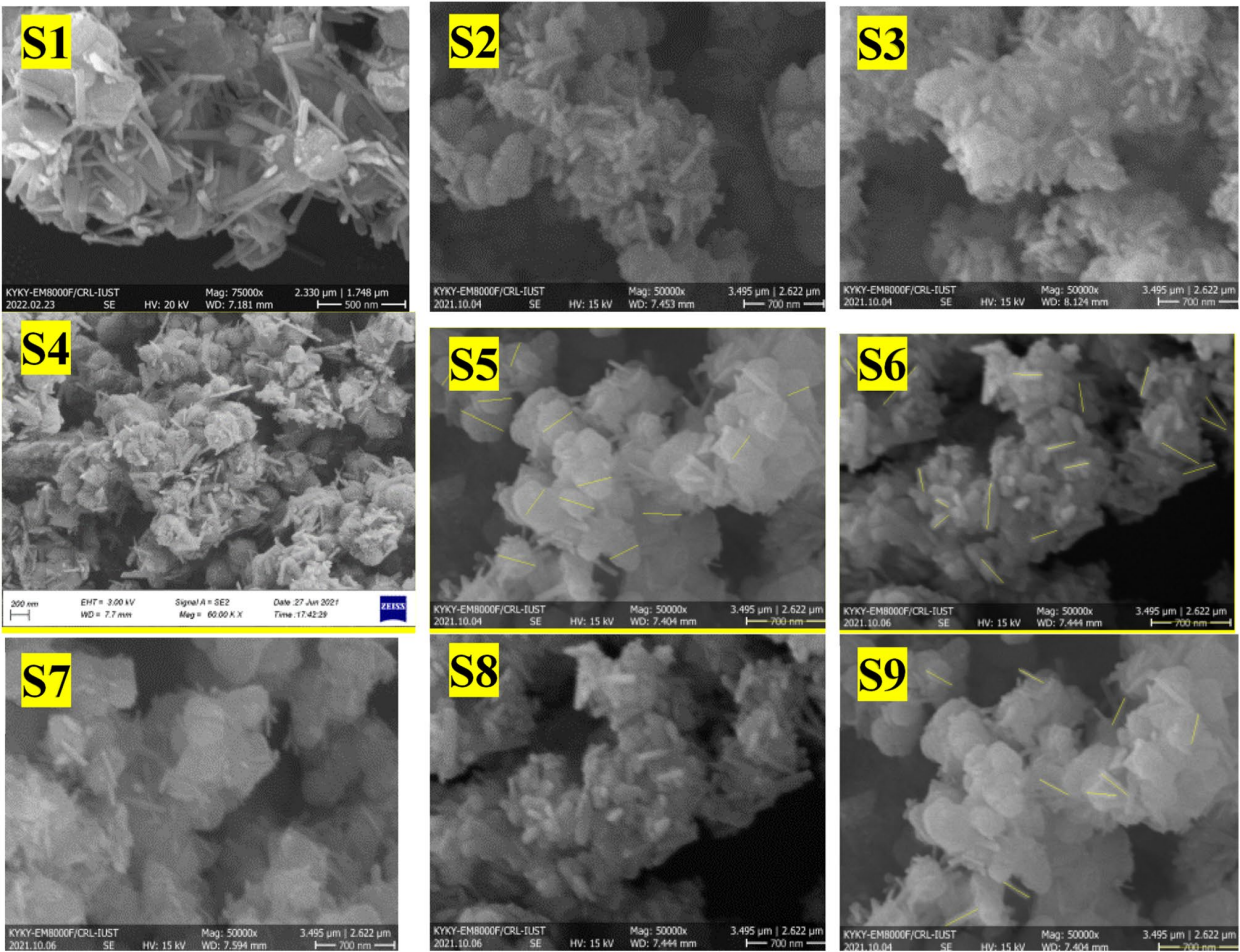
FESEM images of nanourchin-like samples S1–S9.

The surface morphology of Fe–Mn double oxides was inspected by FESEM. As it appeared, Fe–Mn sample S4 consists of invariant nanostructure architectures with a typical breadth of 250 nm (Fig. 6 (S4)) which is smaller than S2 (300–400 nm). Nucleation centers comprising of Fe–Mn double oxides (FeMn_x_O_y_) were formed rapidly by the redoxidation between KMnO_4_ and FeSO_4_, though needle-like was created spirally from interfacial cores^48^. As is well known, morphology is dependent on extrinsic and inborn energy. The extrinsic energy controls growth rates, whereas the inborn property decides the nucleation growth^49^. Compared to S1, S5, and S9, in the same reaction temperature, the number of nanoneedles increases when the concentration of NaOH increases. However, it grows randomly and the diameter-to-length ratio is approximately constant. FESEM of samples S2, S6, and S7 shows that by increasing the temperature the ratio of diameter to length is be smaller. It means that temperature with the higher ratio of FeSO_4_/KMnO_4_ and enough NaOH produced a stable structure with a small diameter and high length nanoneedles. When the reaction temperature is increased to 100 ^◦^C by enough FeSO_4_ and NaOH, sample S4 with the regular structure and the small diameter of nanoneedles (15–20 nm) will be formed. In the study of Zheng et al., the impact of the molar proportion of KMnO_4_ to oleic corrosive on the structure and morphology of urchin-like Mn_3_O_4_ was explored. The nanoneedles on the surface of nanourchin-like particles were deficient, with a width of 20 nm and a length of 70 nm. When the molar proportion was 100:1, the nanoneedles were developed vertically on the surface of nanoparticles^50^. The comparison of current work with different urchin-like nanostructures is listed in Table 7.

**Table 7:** Comparison of nanourchin-like materials.

Name	Diameter (nm)	Length (nm)	D/L	BET (m^2^/g)	References
Urchin-like Mn_3_O_4_	30	150	0.2	198.76	^32^
Sea urchin-like carbon/ZnO	40	147	0.27	64.3	^33^
Urchin NiCoP	20	300	0.06	–	^35^
Urchin NiCO_2_O_4_	18	–		41	^36^
Urchin ZnO	20	–	–	53.9	^51^

**Figure 7:**
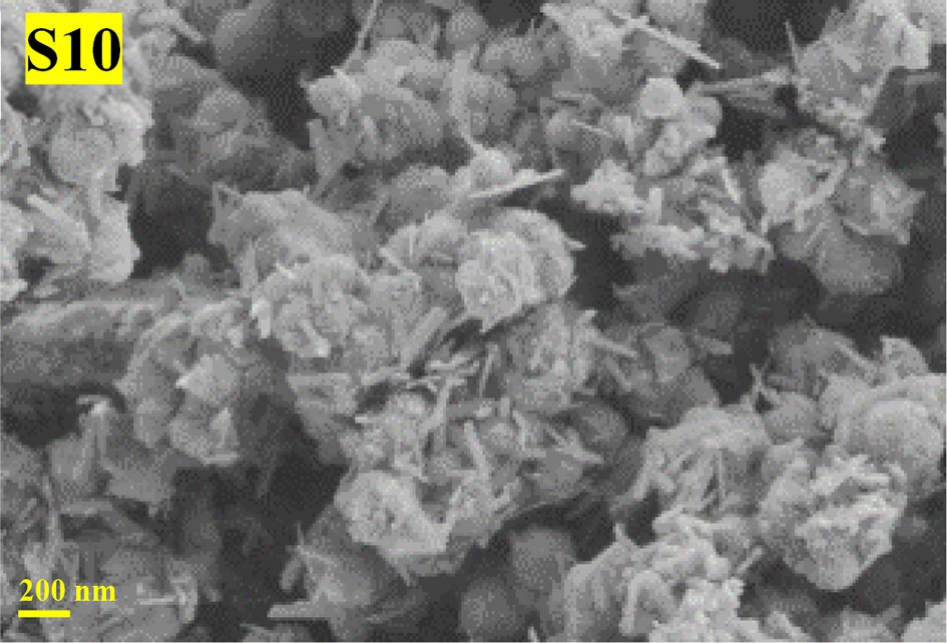
FESEM image of nanourchin-like sample S10.

An assertion test must also be performed to affirm the expected regard underneath the perfect confirmation levels. The test will control the perfect situations propounded. Thus, in extension to 9-synthesized Taguchi design samples, the 10th sample (Fig. 7) was orchestrated underneath the ideal situation. Brough almost D/L and regard was 0.125 showing a small desire error (% 4).

**Figure 8:**
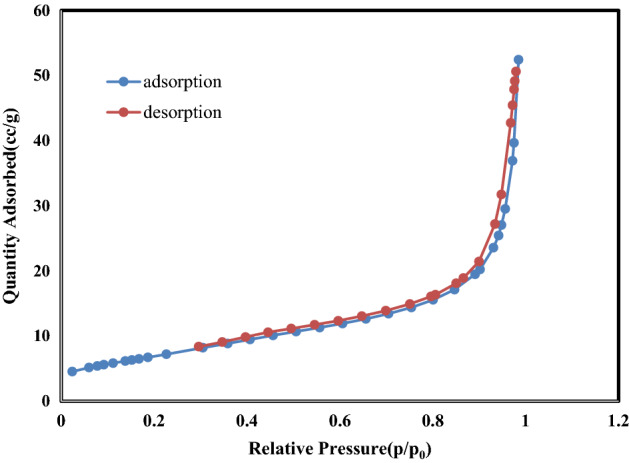
Nitrogen adsorption–desorption isotherms of S3.

**Figure 9:**
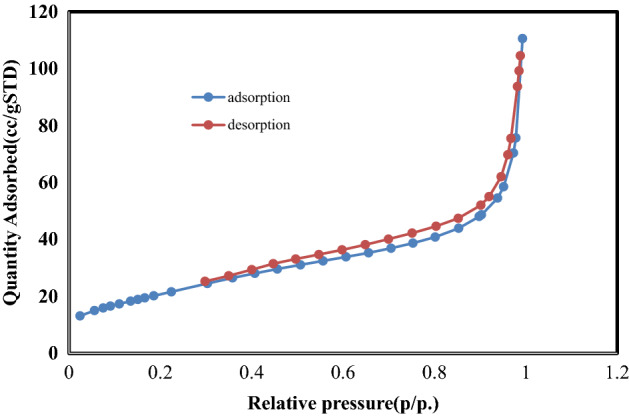
Nitrogen adsorption–desorption isotherms of S4.

The BET results were conducted to further investigate the internal pore structure. The pore size dispensation was derived from the desorption branch of the isotherm with the Barrett–Joyner–Halenda model (Belsorp Mini2-Japon). The result of BET surface analyses confirmed the result of the Taguchi method. S4 has a specific surface area of 74 m^2^/g, much higher than S3 (25 m^2^/g) (Figs. 8 and 9). Furthermore, the pore volume and the average pore size of the S4 and S3 samples are 3 and 8.7 nm and 0.17 and 0.08 cm^3^/g_,_ respectively. The results show that the smaller diameter-to-length ratio has a high specific surface area.

## Conclusion

In summary, urchin-like Fe–Mn double oxides nanostructure was synthesized in a simple one-step precipitation arrangement without encouraging surfactant substances. Tests were conducted utilizing Taguchi L_9_ statistical design. After altering the samples, morphological highlights and chemical investigations such as XRD, FESEM, and EDX were performed. The highest response alteration depended on KMnO_4_ and FeSO_4_.7H_2_O concentration with contribution percent of 32.13 and 30.90%, respectively. The most variables for synthesizing the ideal sample with the lowest ratio of diameter to length were 225 mM of FeSO_4_.7H_2_O solution, 59 mM of KMnO_4_, 5 M NaOH, and the reaction temperature of 85 ^◦^C. The BET surface analyses of samples S3 and S4 showed a correlation between the ratio of D/L and surface area. It meant that the nanourchin with the high length and the small diameter had a high specific surface area.

## Data Availability

Data are available [from Ahmad Rahbar-Kelishami] with the permission of [Ahmad Rahbar-Kelishami]. The data that support the findings of this study are available from the corresponding author, [Ahmad Rahbar-Kelishami], upon reasonable request.
